# Investigating environmental heat exposure and its effects on maternal and fetal health in rural Northern Ghana (Physio-HeMAB Study): a study protocol for a cluster randomized trial

**DOI:** 10.1186/s12889-026-28030-8

**Published:** 2026-07-06

**Authors:** Rachel Sophie Zimmer, Edmund Yeboah, Lila Sax Dos Santos Gomes, Lena Fiebig, Antonio Torres Reyes, Alina Hermann, Aaron Kampim, Callistus Ireneous Nakpih, Annalena Horst, Michael Bergner, Marcus Riemer, Stephanie Wallwiener, Gilbert Abotisem Abiiro, James Akazili, Jan W. Kantelhardt, Martina Anna Maggioni, Manuela De Allegri, Daniel Azongo, Eva J. Kantelhardt

**Affiliations:** 1 Global and Planetary Health Working Group, Martin-Luther-University Halle-Wittenberg, Halle (Saale), Germany; 2 Heidelberg Institute of Global Health, Faculty of Medicine and University Hospital, Heidelberg University, Heidelberg, Germany; 3 Yarrow Global Consulting gGmbH, Staffort, Germany; 4 Navrongo Health Research Center, Research and Development Devision, Ghana Health Service, Navrongo, Ghana; 5 C.K. Tedam University of Technology and Applied Sciences, Navrongo, Ghana; 6 Department of Obstetrics and Prenatal Medicine, University Hospital Halle, Halle (Saale), Germany; 7 University of Development Studies, Tamale, Ghana; 8 Institute of Physics, Martin-Luther-University Halle-Wittenberg, Halle (Saale), Germany; 9 Charité Center for Global Health (CCGH), Charité—Universitätsmedizin Berlin, Berlin, Germany; 10 Department of Biomedical Sciences for Health, Università degli Studi di Milano, Milan, Italy

**Keywords:** Heat, Heat stress, Pregnancy, Physiology, Fetal health, Wearable sensors, Physiological monitoring, Climate change adaptation

## Abstract

**Background:**

Climate change increases the frequency and intensity of heat events. Previous studies have established a correlation between heat exposure and adverse pregnancy outcomes, including preterm birth, stillbirth, and other maternal and fetal health complications. However, evidence on the physiological pathways underlying heat strain during pregnancy and on whether heat adaptation strategies can modify these responses remains limited, particularly in low-resource settings. This study aims to assess the effects of environmental heat exposure on maternal and fetal physiological responses among pregnant women exposed and unexposed to a heat adaptation strategy.

**Methods:**

Physio-HeMAB is an interventional, cluster-randomized study hosted by the Health and Demographic Surveillance System in Navrongo, Ghana. This study integrates: (i) high-resolution environmental monitoring including Wet-Bulb Globe Temperature at household level and Universal Thermal Climate Index environmental data at regional level; (ii) continuous maternal physiological assessment via wearable devices measuring heart rate, heart rate variability, core body temperature, physical activity, and sleep; and (iii) fetal health assessments, including fetal heart rate and Doppler indices. This multimodal framework supports detailed assessment of how varying levels of environmental heat exposure relate to maternal physiological changes and fetal health indicators. Descriptive and multivariable statistical methods will be used to analyze the data.

**Discussion:**

A better understanding of the physiological mechanisms of heat strain during pregnancy, and an evaluation of whether targeted adaptation strategies can mitigate its effects, are essential for protecting maternal and neonatal health. This Physio-HeMAB study integrates individual-level physiological responses to environmental heat exposure and heat adaptation strategies. This approach strengthens the evidence for developing context-specific and gender-responsive public health interventions.

**Trial registration:**

This study was registered in the Pan African Clinical Trials Registry as PACTR202601761543866 on 05 January 2026.

**Supplementary Information:**

The online version contains supplementary material available at 10.1186/s12889-026-28030-8.

## Introduction

The climate crisis is widely recognized as one of the biggest global health threats of the 21st century [1]. Climate change acts as a “threat multiplier”, intensifying existing social, economic, and health inequalities, and disproportionately impacting vulnerable populations [2]. Pregnant women are especially affected due to intersecting structural inequalities and limited access to adaptation resources, particularly in regions with fragile health systems. This highlights the critical connection between climate resilience and gender equality [3, 4].

The increasing frequency and intensity of extreme weather events, particularly heatwaves, is a leading cause of weather-related deaths worldwide and represents one of the most urgent concerns facing the global community today [5–7]. Sub-Saharan Africa (SSA) is particularly vulnerable to the complex effects of climate change [8–11]. Ghana, in West Africa, along with many countries in SSA, has experienced an increase of extremely hot days that threaten maternal and newborn health [12]. According to Climate Central, 75% of the pregnancy-related heat-risk days in Ghana are directly attributable to human-induced climate change [12]. Projections from the World Meteorological Organization indicate that this trend will intensify in the coming years [13].

SSA already accounts for nearly two-thirds of the worldwide maternal deaths annually [14–16], and the region experiences a disproportional share of global preterm births [17]. This trend may worsen, as exposure to extreme heat is strongly associated with adverse pregnancy outcomes. A meta-analysis published by Lakhoo et al. in Nature Medicine reported that higher heat exposure is associated with 29% higher odds of low birth weight compared with lower heat exposure. Similarly, short-term heat exposure was associated with a 4% increase in the odds of preterm birth per 1 °C rise and a 26% increase during heat waves [18]. Notably, late preterm birth has been associated with increased temperature exposure during gestational weeks 34–37, suggesting a critical heat-sensitive period in the final weeks of pregnancy [19]. Lakhoo et al. reported increased odds of stillbirth (OR = 1.13) and gestational diabetes mellitus (OR = 1.28) with high heat exposure, and a 25% increase in obstetric complications during heat waves [18].

The biological nature of pregnancy increases vulnerability to heat-related health risks [18, 20, 21]. Pregnancy involves substantial cardiovascular adaptations required to meet the metabolic and circulatory demands of the growing fetus [22–24]. These adaptations are largely regulated by the maternal autonomic nervous system (ANS). Evidence from Bester et al. suggests that advancing gestation is associated with attenuated autonomic responsiveness and reduced system complexity [22]. This potentially reflects a stable and tightly regulated ANS necessary to maintain physiological balance during pregnancy. However, the reduced autonomic flexibility may limit the maternal capacity to rapidly respond to external environmental stressors such as heat exposure [22]. Pregnancy is accompanied by further physiological and anatomical changes, including greater metabolic heat production due to fetal growth, a higher BMI, and a reduced surface area-to-mass ratio [25]. Therefore, the body’s thermoregulatory capacity to maintain core body temperature (CBT) within the narrow range of 37 ± 0.5 °C could be compromised, making it harder to dissipate heat [25]. During heat exposure, increased sweating (i.e., increased loss of body fluids) and inadequate fluid intake may lead to dehydration. This can cause electrolyte imbalances and endocrine disruption potentially triggering premature onset of labor [21]. Additional pathways, including altered glucose metabolism, inflammatory responses, impaired placental development, and disrupted maternal blood flow are hypothesized to contribute to adverse pregnancy outcomes [21, 22, 26–29].

The use of research-grade wearable devices (“wearables”) and environmental data loggers facilitates detailed assessment of such physiological responses to heat exposure under real-world conditions [30–32]. The feasibility and acceptability of wearable technologies have been demonstrated in rural African settings, supporting their use for longitudinal monitoring during pregnancy [30, 33]. Beyond indicators of the maternal ANS such as heart rate and heart rate variability, wearables can be used to estimate physical activity patterns and sleep characteristics [31, 34–36] both of which are relevant pathways through which heat exposure may affect maternal well-being.

Among pregnant women, heat exposure has, for example, been associated with sleep disturbances, fatigue, and heat-related illness, particularly in contexts of strenuous physical labor and where homes cannot be adequately cooled [37, 38]. These impacts are further intensified by gender norms and gendered divisions of labor, which can constrain the support available to pregnant women within households and mean that unpaid domestic and small-holding farm work is continued, even during periods of extreme heat [37, 39]. Limited social and material resources available to women further compound vulnerability during pregnancy [39, 40].

In Ghana, initial steps have been taken to build a climate-resilient health infrastructure. The National Climate Change Policy identifies “Focus Area 3: Increase resilience of vulnerable communities to climate-related risks”, specifically recognizing women as a vulnerable group and outlining actions to strengthen community resilience [41]. Other Ghanaian policies and plans also aim to integrate climate risks into the health sector. Despite these initial efforts, the implementation of climate change adaptation strategies in policies and national plans remains incomplete, and mainstreaming has been limited by insufficient coordination and information systems, as well as inadequate financing [42]. Although women are recognized as a vulnerable group [41], pregnancy and its unique vulnerabilities are often overlooked by policymakers and program implementers in Ghana, reflecting a broader trend observed across many low-resource settings [20, 34, 35].

This highlights the urgent need for an evidence-based, gender-transformative adaptation strategy. Physio-HeMAB specifically evaluates the physiological maternal and fetal response in individuals exposed and not exposed to such a heat adaptation strategy. To achieve this, it adopts a multi-level data collection design, combining environmental heat exposure measurements, continuous maternal physiological monitoring using wearables, and fetal health assessments in an integrated approach.

## Methods and analysis

### Study objectives

Physio-HeMAB study has the following objectives:

Primary objective: to assess the effect of heat exposure (with and without the heat adaptation strategy) on (1) maternal heart rate as a proxy for maternal heat strain.

Secondary objectives: to assess the effect of heat exposure (with and without the heat adaptation strategy) on (1) maternal heart rate variability and (2) physiological strain index as further proxies for maternal heat strain, (3) maternal sleep duration, (4) maternal physical activity, and (5) on fetal strain during assessments.

### Study setting

This study is set in Ghana, a lower-middle-income country in West Africa, which has a tropical climate and annual mean temperatures between 26 °C in the south and 29 °C in the northeast [43]. More specifically, this study will be conducted in the Upper East Region (UER), in the area covered by the Navrongo Health and Demographic Surveillance System (HDSS), run by the Navrongo Health Research Center (NHRC). It is the hottest part of the country, with temperatures reaching up to 40 °C during the dry season lasting from November until April [44]. Climate data indicates a national average temperature rise by 1 °C, with the greatest rate of change in the UER with increases estimated at 2.0–2.5 C° [45].

The HDSS, which monitors approximately 172,000 individuals (52.3% female) and 38,400 households in the two Kassena-Nankana districts, records approximately 4,000 births annually [46, 47]. A network of Community Key Informants, supported by official birth registration systems, ensures the timely collection of data on pregnancies and births [47].

The study will be conducted during the dry season, when mean monthly daytime temperatures are expected to range between 35.5 °C and 38.8 °C [44].

### Study design

Physio-HeMAB is an interventional, cluster-randomized substudy embedded within the larger parent HeMAB trial (PACTR202510765394928). In the parent trial, the heat adaptation strategy (intervention) is allocated to 16 health centers (HCs) through cluster randomization. The primary outcome of the larger HeMAB study is the change in birth weight, assessed in the 3,200 births annually in the study area (*n* = 1,600 per cluster).

**Fig. 1:**
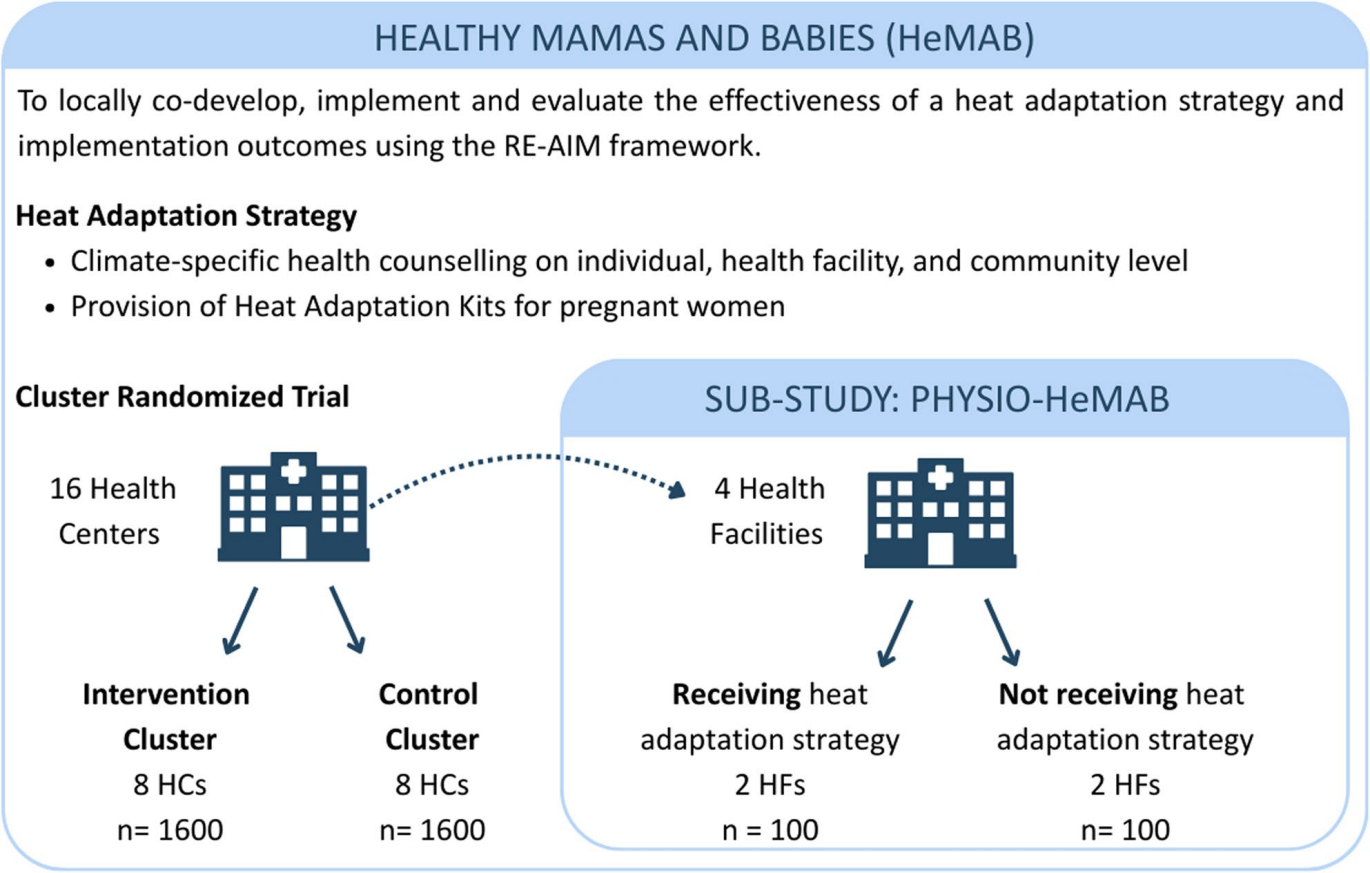
Overview of the Physio-HeMAB study nested within the larger healthy mamas and babies (HeMAB) trial

Physio-HeMAB does not conduct randomization itself; however, it uses the pre-existing randomized allocation within the existing trial framework to evaluate intervention effects on physiological outcomes. Therefore, by definition, Physio-HeMAB constitutes an interventional study with a cluster-randomized design. The HeMAB study design is visualized in Fig. 1.

Within this framework, Physio-HeMAB applies prospective physiological monitoring to assess maternal and fetal responses to environmental heat in both intervention and control clusters. Each Physio-HeMAB cluster comprises two health facilities (one hospital and one affiliated health center), purposefully selected to ensure comprehensive data collection. At the hospital level, permanent gynecologists perform ultrasound examinations, with support from midwifery staff. The associated health centers are operated by trained midwives who deliver essential maternal and reproductive health services.

### Conceptual framework

**Fig. 2:**
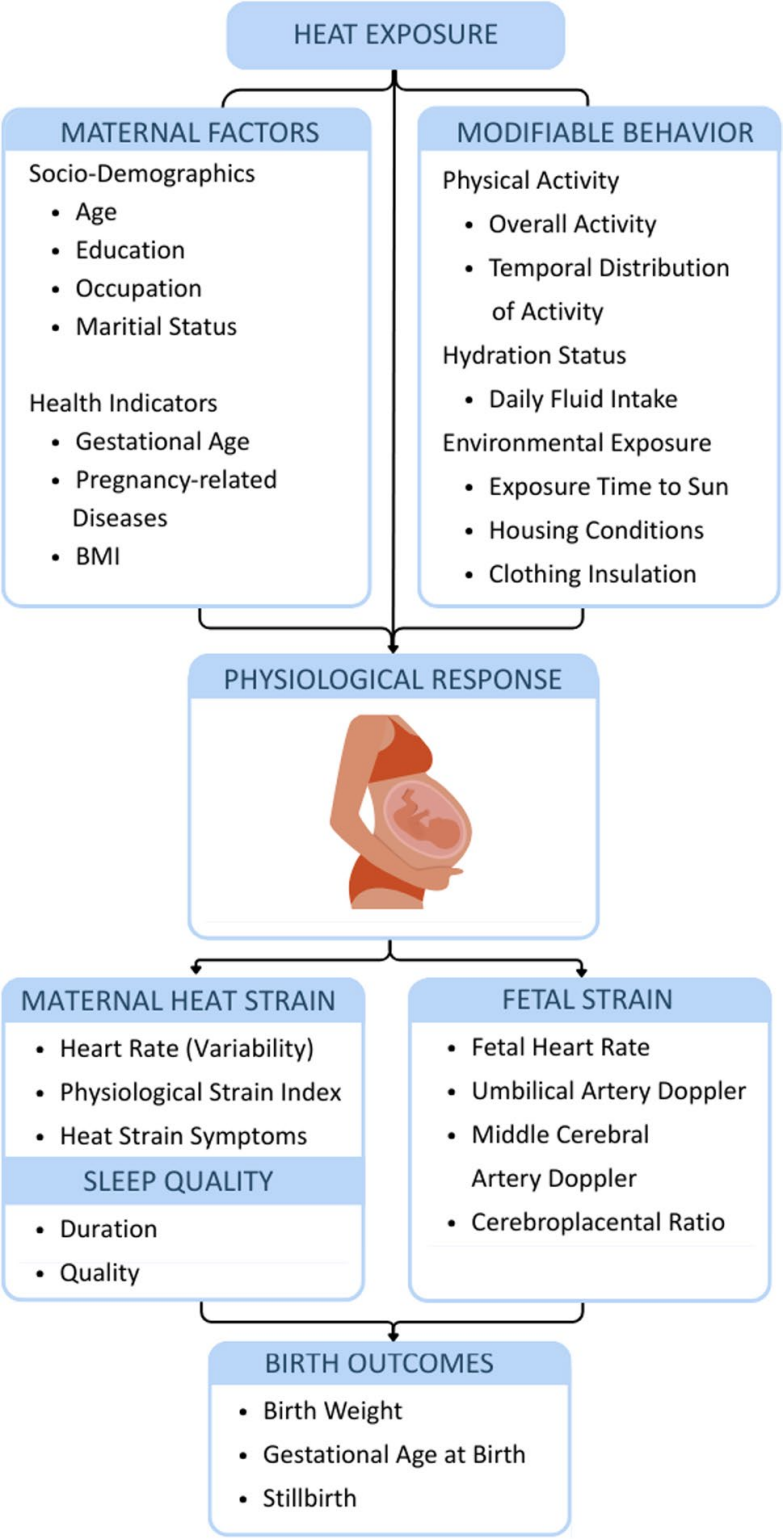
Causal pathway for linking heat exposure, modifiers (maternal factors and behavior) with the physiological response (maternal and fetal) with pregnancy outcomes

The conceptual framework, linking heat exposure, modifying factors, maternal and fetal physiological responses, and pregnancy outcomes, is depicted in Fig. 2. The primary exposure is environmental heat. The extent to which heat impacts health may vary depending on maternal factors (socio-demographic and health-related characteristics) and modifiable behaviors (physical activity, hydration status, and environmental exposure). Together, these characteristics and behaviors influence the physiological response to heat, which in this study is assessed through fetal strain, maternal heat strain and sleep quality. The physiological response could in turn affect birth outcomes such as birth weight, the gestational age at birth and the occurrence of stillbirth.

### Measurement methods

The Physio-HeMAB study aims to provide a comprehensive view of the dynamic interaction between maternal physiology, fetal strain, and environmental heat stress. Therefore, socio-demographic and clinical data will be collected during antenatal care, wearable devices will continuously measure maternal physiological parameters, and environmental sensors will continuously log heat stress.

#### Data obtained at the health care facility

A gynecologist will ascertain gestational age through the last menstrual period or the biparietal diameter, measured by ultrasound. Doppler ultrasound recordings of the umbilical artery, middle cerebral artery, and cerebroplacental ratio; as well as cardiotocography recordings of the fetal heart rate, will also be measured at consultations. During these visits, standard clinical measurements (tympanic temperature, pulse, and blood pressure) will be recorded, and a questionnaire on maternal factors including socio-demographic characteristics and health indicators, will be completed. Questionnaires on daily routines, fluid intake, and sleep logs will be administered to complement the wearable device measurements. Participants’ feedback on the acceptability and perceived burden of the study procedures will be obtained in the closing questionnaire. Birth outcomes will be evaluated post-partum using patient records.

#### Data collected by sensors

Five sensors will collect data over a seven-day study period for each participant (Table 1: Device specifications).

**Table 1:** Device specifications

DEVICE	GENEActiv Original	Bittium Faros™ 180	Calera Research	Hygrometer PCE-WB 20SD	SwitchBot Meter
COMPANY	ActivInsights, United Kingdom	Bittium Biosignals Ltd., Finland	greenTEG AG Switzerland	PCE Instruments UK Ltd., United Kingdom	SWITCHBOT INC, USA
MEASURE-MENT	Three-axial Acceleration, Near body temp., Ambient light	ECG, Acceleration	Estimated CBT, Skin temp., Acceleration	WBGT	Air temp., Humidity
TYPE OF SENSOR	Wearable motion sensor	Biomedical wearable electro-cardiogram	Skin temperature and heat-flux monitor	Environmental heat stress meter	Environmental sensor
RESOLUTION	100 Hz	250 Hz	17 mHz	17 mHz	17 mHz
DATA TRANSFER	Cradle with USB cable	Micro-USB cable	Bluetooth BLE	SSD Card	Mobile Phone Application
DATA FORMAT	BIN	EDF	CSV	CSV	CSV

##### Physiological data

Continuous physiological data will be collected with research-grade wearable devices. These wearables are: (1) an Electrocardiography (ECG) device (Bittium Faros™ 180) to record maternal cardiac electrical activity and derive maternal heart rate (HR) and heart rate variability (HRV). (2) A core body temperature sensor (Calera Research), which derives core body temperature (CBT) from continuous monitoring of skin temperature and heat flux, and (3) an accelerometer (GENEActiv Original) for continuous three-axial accelerometry recordings, to assess physical activity and sleep.

##### Temperature data

Heat and heat stress will be observed at individual and regional levels. On the individual level, participants will receive 4) Hygrometers (PCE-WB 20SD) to be placed outside their homes and measure Wet-Bulb Globe Temperature (WBGT) wherever feasible. 5) Switch Bot Meters (SwitchBot) will record home indoor air temperature and humidity. For regional observations of the Navrongo Upper East Region, retrospective Universal Thermal Climate Index (UTCI) data from the ERA5 data set provided by the Copernicus Climate Data Store [48] will be utilized.

### Measured variables

In alignment with the conceptual framework, exposure, covariables, and outcomes are specified in Table 2.

**Table 2:** Expected outcome categories, relevant outcome measures, variable types, measurement method and level of analysis

CATEGORY	MEASURE	VARIABLE TYPE	MEASUREMENT METHOD
EXPOSURE			
Environmental measurements	WBGT	Continuous	Hygrometer PCE-WB 20SD
	Air temperature and humidity	Continuous	SwitchBot Meter
	UTCI	Continuous	ERA5 data set
COVARIATES/ CONTEXT			
Maternal factors	Socio-demographic factors	Categorical/ Continuous	Questionnaire, Clinical record
	Health indicators	Categorical/ Continuous	Questionnaire, Clinical record, Physical examination
	Modifiable behaviors	Categorical	Questionnaire
Physical activity	Time- and temperature-specific physical activity	Continuous	GENEActiv Original, Hygrometer PCE-WB 20SD, Activity diary
	Domain-specific physical activity	Categorical	Activity diary
Study-related	Intervention status	Binary	Study assignment
OUTCOMES			
Maternal heat strain	HR and HRV Physiological Strain Index	Continuous Continuous	Bittium Faros™ 180 Bittium Faros™ 180, Calera Research
	Self-reported heat strain symptoms	Categorical	Questionnaire
Sleep quality	Sleep duration	Continuous	GENEActiv Original, Self-reported sleep-log
	Self-reported sleep quality	Score	Self-reported sleep-log
Fetal strain	Fetal HR	Continuous	Cardiotocography
	Umbilical artery and middle cerebral artery Doppler parameters	Continuous	Doppler ultrasound
	Cerebroplacental ratio	Continuous	Doppler ultrasound
Neonatal health	Birth weight	Continuous	Clinical record
	Gestational age at birth	Continuous	Clinical record
	Stillbirth	Binary	Clinical record

### Participant timeline

**Fig. 3:**
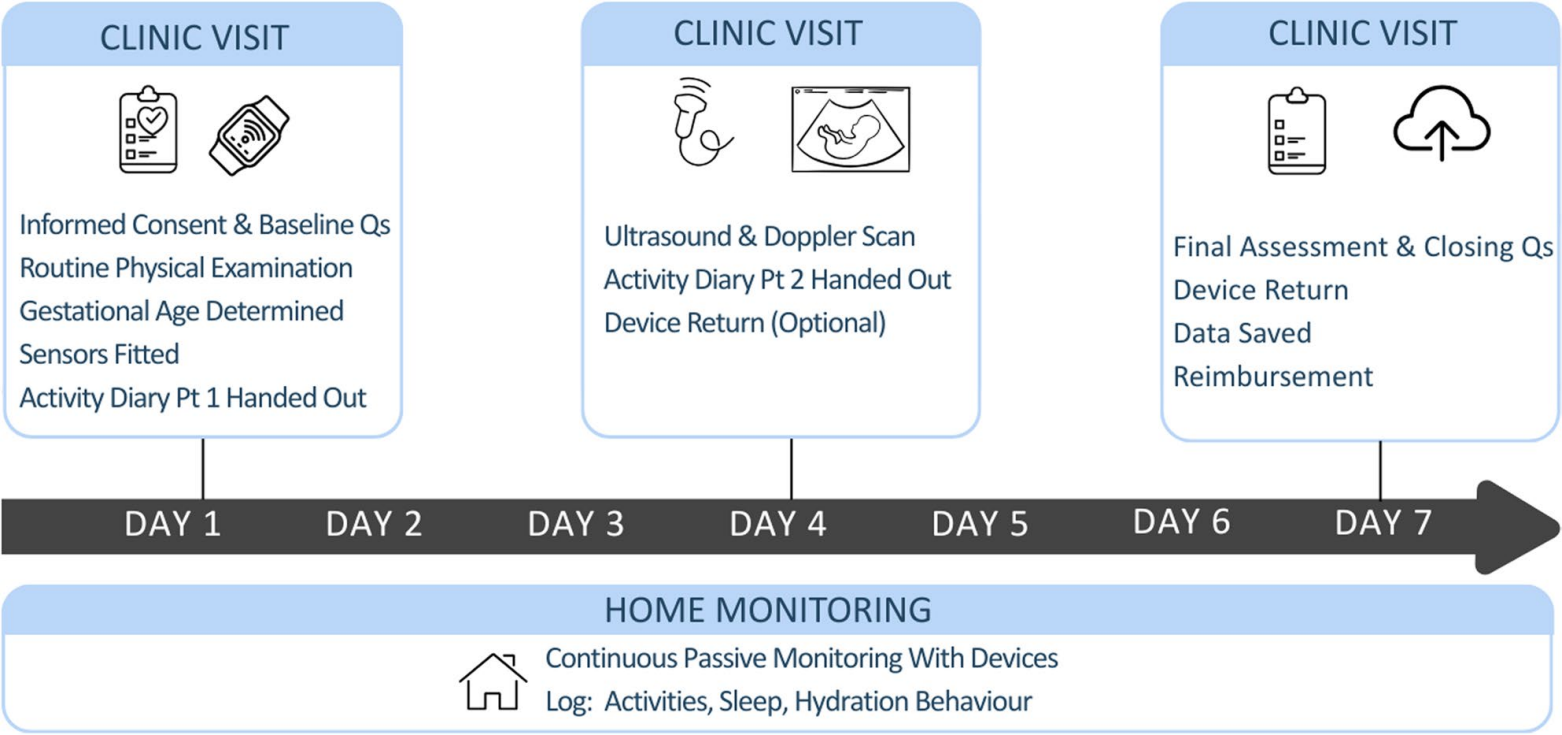
Study timeline. Seven-day continuous monitoring period with three scheduled clinic visits. Pt = Part, Qs= Questionnaires

Participants will be enrolled in a seven-day monitoring period. Physiological and behavioral data will be continuously collected with wearable sensors and a self-logged activity diary. Environmental sensors will measure on individual level throughout the week. Participants visit their health facility on days one, four, and seven. Study procedure details and activity flow are depicted in Fig. 3.

Participants are encouraged to continue all components of data collection throughout the seven-day monitoring period, as this will capture more diverse exposure to environmental stressors. However, participants are given the option to terminate the trial on day four, in which case final study procedures will be conducted on day four instead of day seven.

### Sample size and selection of subjects

The sampling method will be consecutive sampling of women who visit any of the four purposefully selected HDSS health facilities for antenatal consultations. Consideration will be made for pregnant women who can conveniently attend the health facility at least three times during the physiological measurement period. Inclusion criteria are (1) being a pregnant woman in the third trimester (> 28 weeks) with a (2) singleton pregnancy. Further inclusion criteria are (3) being of legal age, (4) able to give consent, (5) registration (having an ANC card) and (6) living within 10 km proximity of one of the four selected HDSS health facilities. Exclusion criteria are (1) illness that requires hospital admission or immediate delivery, (2) known fetal malformations, and 2) a known psychological or mental health concern.

The primary outcome variable is maternal heart rate (beats per minute, bpm), which serves as a physiological proxy for maternal heat strain, reflecting cardiovascular responses to heat exposure. To determine an appropriate sample size for the Physio-HeMAB study, a power analysis was conducted for comparing mean heart rates between two independent groups. Assuming a mean detectable difference of 5 bpm, a common standard deviation of 10 bpm, a two-sided significance level (α) of 0.05, and 80% statistical power, the required sample size is 63 per group. The study will enroll 200 participants in total across the four HDSS health facilities, 100 in the intervention group and 100 in the control group. As this is the first time this type of study will be conducted in this setting, it is not yet possible to precisely estimate potential attrition, data loss and inter-individual variability. Therefore, the sample size is chosen to provide a conservative margin, ensuring that even with 30% attrition, the study retains adequate power to detect clinically relevant differences in heart rate between groups.

Participants will be invited to provide feedback on the acceptability and perceived burden of study procedures as part of the Patient and Public Involvement strategy, to help refine future implementation and promote inclusive participation that builds trust in the research institution. To promote participant retention, participants will benefit from enhanced assessments of well-being during pregnancy, including an additional ultrasound scan. In the case of incidental findings, participants who choose to be informed will be referred for additional diagnostic evaluations. The study team will cover transportation and treatment costs in accordance with local standards, and participants will receive soap and refreshments.

### Statistical methods

To evaluate potential effects of heat exposure and the heat adaptation strategy on fetal and maternal physiological responses, behavioral adaptations, and pregnancy outcomes, a multi-level analytical framework will be employed. Descriptive analyses will summarize each study variable, presenting measures of central tendency (e.g., mean, median) and variability (e.g., standard deviation, interquartile range) for continuous variables, and absolute and relative frequencies for categorical variables. Subgroup comparisons by intervention status, exposure to heat-risk periods during pregnancy, age group, gestational age, and occupation will be performed to indicate group-specific patterns in heat-related effects.

Both univariable and multivariable analyses will be used to explore preliminary associations between exposure, mediators, and outcomes. For repeated measurements Linear mixed effects models (LMMs) with random intercepts will be fitted (e.g., heart rate, HRV, physical activity), incorporating both time-varying covariates (e.g., WBGT, time of day) and fixed participant-level covariates (e.g., age, BMI, gestational age, parity, occupation). Interaction terms between intervention and heat exposure will be tested to assess potential effect modification. Non-linear extension will be considered if necessary.

### Data safety and monitoring

Continuous data recordings in this study include maternal ECG, triaxial accelerometry data, CBT, as well as WBGT measurements, ambient temperature and humidity data. All physiological and environmental data will be continuously recorded over a seven-day study period and temporarily stored on the respective devices. Subsequently, the data will be pseudonymized, removing any personal identifiers, and securely exported to encrypted, password-protected study computers. In parallel, the datasets will be backed up to two encrypted external hard drives to ensure redundancy and data safety. Data will then be securely transferred to Martin-Luther-University local computers by the external hard drives. Analysis will only be conducted on secure study computers. There is no ancillary and post-trial care as no harm from the trial participation is expected. Yet, appointed principal investigators will have access to interim results and the decision-making competencies to terminate the trial.

### Auditing

To ensure high data quality and protocol adherence, regular internal audits will be conducted by a member of the study team who is not involved in day-to-day data collection. These audits will include random checks of data completeness, consistency, and accuracy in the electronic database, with particular focus on physiological variables (e.g., HR, HRV, CBT, accelerometry data). Additionally, quality checks will be performed for data collected with the sensors to detect potential signal loss, noise, device malfunction, or non-physiological spikes, ensuring that only valid and reliable measurements are included in the analyses. Following the first data-collection phase and throughout the data-collection process, feedback sessions will be held among all data collectors, on-site researchers, and implementers to inform subsequent data collections.

### Harms and benefits

The measurements conducted during this study are non-invasive, and according to the manufacturers, do not pose a risk when worn [49–51]. Participants may benefit from additional assessments of their well-being during pregnancy and can choose to be informed about any incidental findings. These findings may be similar to those typically detected during routine antenatal care, such as maternal or fetal tachycardia, fetal growth restriction, fetal malformations, elevated umbilical artery resistance, or hypertension.

### Dissemination policy

Results from Physio-HeMAB will be integrated into the parent HeMAB study to inform interest-holder engagement and guide gender-transformative approaches with women, community members, healthcare providers, and policymakers. Study findings will be disseminated through peer-reviewed scientific publications, policy briefs, and academic conferences.

## Discussion

Physio-HeMAB is a comprehensive, non-invasive, multi-level physiological assessment designed to capture the complex interplay between environmental heat exposure, maternal physiological responses, and fetal health during pregnancy. A key strength of the study is the integration of continuous environmental monitoring, wearable-based maternal physiological measurements, and fetal assessments within a single analytic framework. Conducting the study within the Navrongo HDSS enables data collection under real-world living and working conditions, enhancing ecological validity and policy relevance. As a sub-study embedded within the larger HeMAB parent trial, Physio-HeMAB leverages the cluster-randomized implementation of a gender-transformative heat adaptation strategy to compare physiological responses between exposed and unexposed participants. This design strengthens the study’s relevance for adaptation policy by linking physiological evidence to a real-world public health intervention. Importantly, the study addresses a critical evidence gap, as most existing heat-related interventions and early warning systems are not tailored to pregnant women, and physiological evaluations of adaptation strategies in this population remain scarce.

Limitations include that the seven-day monitoring period per participant may not fully capture longer-term variability in heat exposure, behavioral adaptation, or physiological responses. Operationally, Physio-HeMAB is the first study of its kind conducted in this specific setting and with pregnant women. As such, uncertainties remain regarding participant retention, data completeness, inter-individual variability, and the performance of the multiple wearables and environmental sensors under field conditions. To address these challenges, the study deliberately enrolls a larger sample than required for the primary outcome, implements retention-enhancing procedures, and applies rigorous data quality control protocols.

Physio-HeMAB is designed to generate valuable methodological and physiological insights. By linking individual-level physiological responses to environmental heat exposure and adaptation strategies, the study supports the evidence base for the development of context-specific, gender-responsive, and scalable public health interventions aimed at protecting maternal and fetal health in a warming climate.

## Supplementary Information

Supplementary Material 1.

## Data Availability

No datasets were generated or analysed during the current study.

## Abbreviations

ANC: Antenatal care
ANS: Autonomic nervous system
BMI: Body mass index
bpm: Beats per minute
CBT: Core body temperature
ECG: Electrocardiography
GDPR: General data protection regulation
GLOHRA: German Alliance for Global Health Research
HC: Health center
HF: Health facility
HDSS: Health and demographic surveillance system
HeMAB: Healthy Mamas and Babies
HR: Heart rate
HRV: Heat rate variability
LMM: Linear mixed effects models
NHRC: Navrongo Health Research Center
SSA: Sub-Sahara Africa
UER: Upper East Region
UTCI: Universal Thermal Climate Index
WBGT: Wet-Bulb Globe Temperature
